
JOURNAL INFORMATION
==============================
NLM Title Abbreviation: Biospektrum (Heidelb)
Journal ID: Biospektrum (Heidelb)
NLM Journal ID: 9615685

Biospektrum

ISSN: 0947-0867
EISSN: 1868-6249

ARTICLE INFORMATION
==============================
PMCID: PMC9425812
DOI: 10.1007/s12268-022-1818-2
Article ID: 1818
Article version: 1
Subjects: Köpfe & Konzepte

Buchrezension zu: Das Virus

Das Virus Auf der Suche nach dem Ursprung von Covid-19, Günter Theißen, 192 S, Westend, 2022. SC, 20,- €., ISBN: 9783864893728, Auch als E-Book erhältlich

Müller Jörg Joerg.mueller@med.uni-jena.de

grid.275559.9 0000 0000 8517 6224 Universitätsklinikum Jena, Jena, Deutschland
Electronic publication date: 2022 Aug 30
Print publication date: 2022
Volume: 28
Issue: 5
First page: 570
Last page: 570
Copyright: © Der Autor 2022
License: Dieser Artikel wird unter der Creative Commons Namensnennung 4.0 International Lizenz veröffentlicht, welche die Nutzung, Vervielfältigung, Bearbeitung, Verbreitung und Wiedergabe in jeglichem Medium und Format erlaubt, sofern Sie den/die ursprünglichen Autor(en) und die Quelle ordnungsgemäß nennen, einen Link zur Creative Commons Lizenz beifügen und angeben, ob Änderungen vorgenommen wurden. Die in diesem Artikel enthaltenen Bilder und sonstiges Drittmaterial unterliegen ebenfalls der genannten Creative Commons Lizenz, sofern sich aus der Abbildungslegende nichts anderes ergibt. Sofern das betreffende Material nicht unter der genannten Creative Commons Lizenz steht und die betreffende Handlung nicht nach gesetzlichen Vorschriften erlaubt ist, ist für die oben aufgeführten Weiterverwendungen des Materials die Einwilligung des jeweiligen Rechteinhabers einzuholen. Weitere Details zur Lizenz entnehmen Sie bitte der Lizenzinformation auf http://creativecommons.org/licenses/by/4.0/deed.de.
License URL: https://creativecommons.org/licenses/by/4.0/

issue-copyright-statement: © Der/die Autor(en), exklusiv lizenziert an Springer-Verlag GmbH Deutschland, ein Teil von Springer Nature 2022

==============================
Diese Rezension erscheint Open Access*

